# Integrated Phenotypic and Sequencing-Based Resistome Assessment of Antimicrobial Resistance Determinants in a Sample of Commercial Farm-Animal Probiotic Products

**DOI:** 10.3390/antibiotics15060544

**Published:** 2026-05-29

**Authors:** Ádám Kerek, Levente Hunor Husz, Edit Szarka, Gergely Álmos Tornyos, Ákos Jerzsele

**Affiliations:** 1 Department of Pharmacology and Toxicology, University of Veterinary Medicine Budapest, István utca 2, H-1078 Budapest, Hungary; husz.levente.hunor@student.univet.hu (L.H.H.); szarka.edit@student.univet.hu (E.S.); tornyos.gergely.almos@student.univet.hu (G.Á.T.); jerzsele.akos@univet.hu (Á.J.); 2 National Laboratory of Infectious Animal Diseases, Antimicrobial Resistance, Veterinary Public Health and Food Chain Safety, University of Veterinary Medicine Budapest, István utca 2, H-1078 Budapest, Hungary

**Keywords:** antimicrobial resistance, probiotics, feed additives, resistome, mobilome, minimum inhibitory concentration, resistome profiling

## Abstract

**Background/Objectives**: Probiotic feed additives are increasingly used in livestock production as antimicrobial-sparing tools, yet viable microbial products should not introduce clinically relevant antimicrobial resistance genes (ARGs) into the intestinal resistome. This study evaluated farm-animal probiotic products using an integrated phenotypic, metagenomic and mobilome-aware safety framework. **Methods**: Seven commercially available products intended for poultry, pigs, cattle or horses were assessed using product metadata, culture-based recovery, broth microdilution minimum inhibitory concentration (MIC) profiling and Illumina short-read sequencing as a screening-level resistome approach. Reads were quality controlled, assembled, screened using the Comprehensive Antibiotic Research Database (CARD)/Resistance Gene Identifier (RGI) workflow and interrogated for plasmid-, phage- and insertion sequence/mobile genetic element-associated genomic context. **Results**: MIC profiles were generated for viable bacterial isolates representing *Enterococcus faecium*, *Pediococcus acidilactici*, *Pediococcus pentosaceus* and *Bacillus subtilis*. One labelled *Lactobacillus plantarum* component was not recovered as viable culture, and one labelled *P. acidilactici* component was recorded as *P. pentosaceus*. Sequencing-based resistome screening identified 30 antimicrobial resistance (AMR)-associated CARD antibiotic-resistant organism (ARO) hits belonging to 13 determinants across six ARG-positive coded products, while one coded product had no retained CARD/RGI hit. Profiles were dominated by recurrent *Enterococcus*-associated background determinants, including *aac(6′)-Ii*, *msrC* and *eatAv*. Plasmid prediction was positive for five hits, whereas no iMGE- or phage-associated ARG context was detected. No *vanA/vanB*, *mcr*, *optrA*, *poxtA*, *cfr*, extended-spectrum β-lactamase (ESBL) or carbapenemase gene was detected. **Conclusions**: The investigated products did not show evidence of high-priority mobile ARG carriage. Nevertheless, AMR-associated determinants and occasional predicted mobile contexts support routine integrated MIC-sequencing-based resistome–mobilome assessment of veterinary probiotic products. Because short-read assemblies do not fully resolve plasmid architecture or transferability, mobile-context predictions should be considered screening-level indicators requiring confirmatory long-read or functional testing for higher-priority findings.

## 1. Introduction

Antimicrobial resistance (AMR) is a major One Health challenge because resistance determinants can circulate across humans, animals, food systems and environmental reservoirs. Wildlife has also been recognized as a potential reservoir and disseminator of AMR bacteria and resistance determinants, further reinforcing the ecological breadth of the One Health AMR problem [1]. In livestock production, antimicrobial-sparing interventions are therefore increasingly promoted, including improved biosecurity, vaccination, optimized husbandry, antimicrobial peptides and microbiome-targeted approaches such as probiotics [2,3,4,5]. Probiotics are generally selected for beneficial effects on host performance, gut stability and pathogen exclusion, but they are living biological products and therefore require safety assessment beyond efficacy alone [6]. Recent Hungarian data also indicate that antimicrobial resistance remains a relevant concern in intensive poultry production systems, supporting the need for antimicrobial-sparing strategies and safety-controlled microbial feed additives [7]. Recent poultry-origin bacterial studies from the region also underline the continued relevance of AMR and virulence-marker surveillance in veterinary microbiology [8].

In the European Union, viable microorganisms intended for use as feed additives are evaluated under a regulatory framework in which taxonomic identity, antimicrobial susceptibility and genomic safety are central components of risk assessment. European Food Safety Authority (EFSA) guidance states that microbial feed additives should not add to the pool of AMR genes in the gut bacterial population or increase the risk of antimicrobial resistance genes (ARG) transfer, and strain characterization increasingly relies on whole-genome sequence-based analysis together with phenotypic susceptibility testing [9,10].

The safety interpretation of AMR in probiotics is not straightforward. Some determinants reflect intrinsic or species-associated background resistance, whereas others are acquired, horizontally transferable and clinically relevant. This distinction is essential because the mere presence of an AMR-associated database hit does not necessarily imply public-health risk. Intrinsic determinants in *Enterococcus faecium*, for example, must be interpreted differently from mobile glycopeptide, oxazolidinone, colistin, extended-spectrum β-lactamase (ESBL) or carbapenemase genes [11,12,13,14].

Previous studies have shown that commercial probiotic products may contain ARGs or transferable resistance determinants, although the level of risk varies substantially among product types, bacterial taxa and analytical pipelines [15,16,17,18,19,20]. Although recent Hungarian veterinary studies have increasingly applied resistome-oriented approaches to livestock-associated bacteria, data on farm-animal probiotic products from Central and Eastern European markets remain limited [21]. The objective of this study was therefore to perform an integrated phenotypic and sequencing-based resistome safety assessment of a targeted sample of commercially available farm-animal probiotic products, combining product metadata, minimum inhibitory concentration (MIC) profiling, resistome analysis and mobilome-aware risk interpretation. The study was not designed as a statistically representative market-wide prevalence survey, but as an exploratory safety assessment of selected product types available in the Hungarian/EU veterinary market.

## 2. Results

### 2.1. Product Composition and Culture-Based Recovery

Seven commercially available farm-animal probiotic products were included, covering products marketed for poultry, swine, cattle or horses (Table 1). Products containing only fungal or yeast-based probiotic components were excluded from the final bacterial AMR/resistome analysis. Label-declared bacterial components included *Enterococcus faecium*, *Pediococcus acidilactici*, *Bacillus subtilis* and *Lactobacillus plantarum*; one included product also contained *Saccharomyces cerevisiae* as a non-bacterial component. Culture-based recovery generated phenotypic MIC profiles for viable bacterial isolates assigned to *E. faecium*, *P. acidilactici*, *P. pentosaceus* and *B. subtilis*. Under the applied culture and recovery conditions, one labelled *L. plantarum* component in Product C was not recovered as viable culture; this finding should be interpreted cautiously because viability may be influenced by product formulation, batch-specific factors and pre-purchase storage conditions. The subsequent sequencing-based analysis was therefore interpreted as a short-read, screening-level resistome assessment of product-derived assemblies rather than as definitive resolution of complete chromosomes, plasmids or transferable genetic elements.

### 2.2. Phenotypic Antimicrobial Susceptibility Profiles

**Figure 1:**
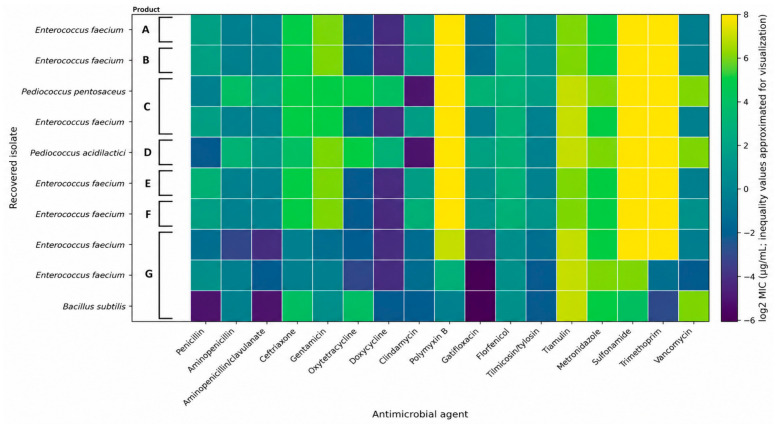
Phenotypic antimicrobial susceptibility profiles of cultured bacterial isolates recovered from farm-animal probiotic products. Minimum inhibitory concentration (MIC) values are visualized on a log2 scale. Inequality values were approximated for visualization only.

Broth microdilution MIC profiling was performed for recovered viable bacterial isolates across a broad veterinary antimicrobial panel (Figure 1; Table 2). The observed profiles were taxon-dependent. The *E. faecium* isolates generally showed low aminopenicillin MICs and vancomycin MICs of 1–2 µg/mL, while high MIC values were observed for cephalosporins, polymyxins, metronidazole, sulfonamide and trimethoprim. These patterns should not automatically be interpreted as acquired resistance because several agents have limited intrinsic relevance for enterococci and should primarily be regarded as phenotypic profiling markers. The *Pediococcus* isolates showed elevated MICs for tetracyclines, aminoglycosides and vancomycin, further supporting the need for genus-specific interpretation. Where EFSA/Feed Edaditives and Products or Substances (FEEDAP) microbiological cut-off values were applicable to the respective probiotic-associated taxon, MIC values were interpreted as safety-screening indicators rather than as clinical breakpoints. Clinical and Laboratory Standards Institute (CLSI) and European Committee on Antimicrobial Susceptibility Testing (EUCAST) clinical breakpoints were not applied as primary interpretive criteria because most organism–drug combinations in this probiotic dataset are not covered by host- and disease-specific clinical breakpoint systems. Therefore, MIC values above a relevant microbiological cut-off were considered decreased-susceptibility signals requiring genomic-context evaluation, whereas elevated MICs for intrinsically non-informative organism–drug combinations were not interpreted as evidence of acquired resistance in the absence of corresponding acquired or mobile ARGs.

### 2.3. Resistome Composition

**Figure 2:**
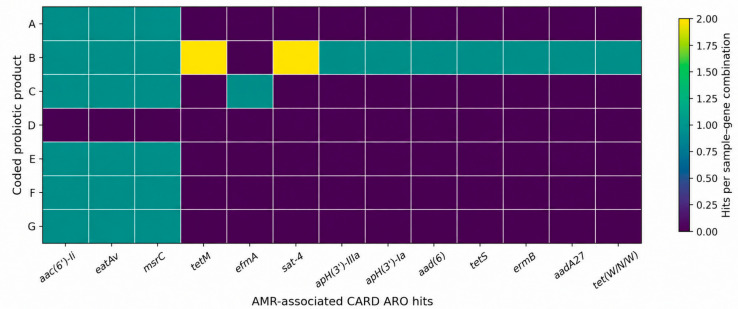
Distribution of the most frequent antimicrobial resistance (AMR)-associated CARD antibiotic-resistant organism (ARO) hits across coded probiotic products. Colour intensity indicates the number of hits per product–gene combination. Product D had no retained CARD/RGI antimicrobial resistance gene (ARG) hit.

Sequencing-based resistome screening of the final coded product dataset identified 30 AMR-associated Comprehensive Antibiotic Research Database (CARD) antibiotic-resistant organism (ARO) hits corresponding to 13 different determinants (Figure 2; Table 3, Supplementary Table S1). ARG-associated hits were detected in six coded products, whereas Product D had no retained CARD/Resistance Gene Identifier (RGI) hit. The most frequent determinants were *aac(6′)-Ii*, *eatAv* and *msrC*, each detected in six products, followed by *tetM* and *sat-4*, each detected twice. The remaining determinants—*aadA27*, *tetS*, *ermB*, *aad(6)*, *aph(3′)-IIIa*, *aph(3′)-Ia*, *tet(W/N/W)* and *efmA*—were detected once. Taxonomic assignment of hit-containing contigs was dominated by *Enterococcus* (19 hits), followed by *Staphylococcus* and *Streptococcus* (three hits each), with single or low-frequency assignments to *Acinetobacter*, Bacilli and broader bacterial categories.

### 2.4. Mobilome-Aware Interpretation

**Figure 3:**
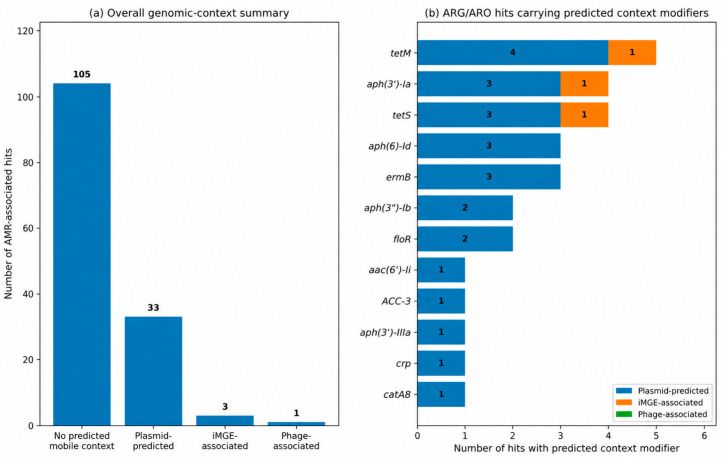
Mobilome-aware classification of antimicrobial resistance (AMR)-associated CARD antibiotic-resistant organism (ARO) hits in the final coded product dataset. (**a**) Overall distribution of AMR-associated hits according to predicted genomic context, distinguishing hits without predicted mobile context from plasmid-predicted, insertion sequence/mobile genetic element (iMGE)-associated and phage-associated hits. (**b**) Gene-level comparison of total AMR-associated hits and hits assigned to predicted mobile contexts. Bar colours indicate the predicted genomic-context category, and bar height indicates the number of hits in each category. Plasmid prediction was interpreted as a risk modifier and not as definitive evidence of horizontal transfer. No iMGE- or phage-associated antimicrobial resistance gene (ARG) context was detected.

Most AMR-associated hits were not predicted to be plasmid-associated. Plasmid prediction was positive for 5/30 hits (16.7%), whereas no iMGE- or phage-associated ARG context was detected in the final coded product dataset (Figure 3). The plasmid-predicted set included tetracycline-, aminoglycoside- and macrolide/lincosamide-associated determinants. Importantly, the dataset did not contain high-priority resistance determinants such as *vanA*, *vanB*, *mcr* genes, *cfr*, *optrA*, *poxtA*, ESBL genes or carbapenemase genes.

## 3. Discussion

The present study places farm-animal probiotic products into the same risk-assessment framework that has recently been applied to human, food-chain and companion-animal probiotic products. The main finding is not the complete absence of AMR-associated determinants, but the absence of high-priority acquired ARGs of major clinical concern, including transferable vancomycin resistance genes, mobile colistin resistance genes, oxazolidinone resistance genes, ESBL genes and carbapenemase determinants. This distinction is important because previous studies have shown that probiotic products and probiotic-associated taxa may carry detectable ARGs, but that public-health relevance depends on taxon identity, gene function, genomic context and mobility potential rather than on ARG counts alone [15,16,17,18,22,23]. In this respect, the present farm-animal dataset appears less concerning than several reports on broader probiotic or food-associated bacterial collections, where ARGs were frequently detected together with plasmid or iMGE signals [16,17].

The results are directly aligned with the EFSA/FEEDAP safety concept for viable microorganisms used as feed additives. EFSA requires that microbial feed additives should not contribute to the intestinal pool of AMR genes and that antimicrobial susceptibility testing should be interpreted together with whole-genome sequence information where decreased susceptibility is detected [9,10]. Our findings support that principle. Phenotypic MIC profiling alone would have overemphasized some elevated values, whereas sequence-only reporting would have overemphasized recurrent background determinants. This is consistent with recent Hungarian veterinary literature emphasizing that phenotypic and genotypic AMR data should be interpreted together because each approach has specific strengths and limitations [24]. The combined MIC-sequencing-based resistome–mobilome assessment therefore provides a more biologically defensible interpretation than either method alone. In practical terms, this means that MIC values exceeding a relevant microbiological cut-off should trigger further genomic and mobilome-context evaluation but should not be reported as clinically resistant phenotypes unless an appropriate clinical breakpoint exists for the specific taxon, antimicrobial and host context. Recent veterinary susceptibility studies across host species also illustrate that phenotypic AMR profiles require host-, taxon- and antimicrobial-class-specific interpretation rather than simple extrapolation across bacterial groups [25].

The predominance of *Enterococcus faecium*-associated *aac(6′)-Ii*, *msrC*, *eatAv* and *efmA*-like signals should be interpreted with particular care. Similar gene sets have repeatedly been described in *E. faecium* strains evaluated for probiotic or food-use safety, where *aac(6′)-Ii*, *msrC* and *efmA* are often regarded as species-associated, chromosomal or intrinsic/background determinants rather than immediate evidence of transferable resistance [23,26,27,28]. Urshev and Yungareva reported that *aac(6′)-Ii*, *msrC* and *efmA* in *E. faecium* LBB.E81 belonged to intrinsic non-transferable factors contributing to lower susceptibility to some antimicrobials, while the strain remained acceptable in an initial safety assessment [27]. Similarly, Xiao et al. detected *aac(6′)-Ii* and *msrC* in a candidate *E. faecium* probiotic strain without major clinical virulence determinants [28]. These observations support our interpretation that recurrent *Enterococcus*-associated background genes should not be treated as equivalent to acquired mobile ARGs. At the same time, *Enterococcus* remains a clinically relevant opportunistic genus; therefore, the absence of high-priority genes such as *vanA* and *vanB* is a critical safety-relevant finding rather than a minor negative result.

The absence of transferable vancomycin resistance genes is especially relevant because several investigated products contained or declared *Enterococcus* components. Vancomycin-resistant enterococci are a major One Health concern, and targeted screening of commercial veterinary probiotics containing enterococci has previously been proposed because such products may be used in animals exposed to intensive antimicrobial selection pressure. Berreta et al. specifically evaluated commercial veterinary probiotic products containing enterococci for *vanA* and *vanB* and emphasized the relevance of monitoring these genes in animal products [19]. Our dataset extends this question from targeted *vanA*/*vanB* screening to a broader metagenomic or resistome framework and similarly did not identify *vanA* or *vanB*. This strengthens the conclusion that the analyzed products did not represent an obvious glycopeptide-resistance hazard, although it does not eliminate the need for continued monitoring.

The lactic-acid-bacterial component of the dataset also requires a cautious, taxon-aware interpretation. Elevated MICs among lactobacilli, pediococci or related LAB should not automatically be equated with acquired resistance, because MIC distributions, incubation conditions, medium composition and EFSA cut-off values may not fully capture species-level biological variation. Recent work on Lactobacillaceae showed that many strains may be classified as aminoglycoside-resistant under EFSA cut-offs while lacking corresponding resistance genes, and the authors argued that genus- or species-level refinement of cut-off interpretation is needed [29]. This is directly relevant to the present study: phenotypic values that appear high for some compounds should be evaluated against species identity and genomic findings, not translated mechanically into clinical resistance categories. This point is particularly important for probiotic safety assessment because false-positive phenotypic classification may lead to exaggerated risk statements, whereas ignoring phenotypic outliers would also be inappropriate.

The *Bacillus*-related findings are likewise best interpreted in comparison with the broader *Bacillus* probiotic literature. *Bacillus* species are common in feed additives because of their spore-forming capacity and stability, but several studies have emphasized that species-level identity and safety screening are indispensable, particularly to exclude members of the *Bacillus cereus* group and strains carrying virulence or transferable resistance determinants [30,31]. Adimpong et al. showed that antimicrobial susceptibility among *Bacillus* strains can be species dependent and that some resistance phenotypes or resistance genes may be present in food-associated *Bacillus* populations [30]. More recently, Jin et al. reported *Bacillus* isolates from probiotic preparations with phenotypic resistance, multiple resistance genes and virulence-associated features, with particular concern for *Bacillus cereus* [31]. Compared with these reports, the present dataset did not show a *Bacillus*-dominated high-risk ARG pattern, but the comparison reinforces why *Bacillus*-containing veterinary products should not be exempt from genomic safety screening.

The mobilome-aware component is the decisive layer of interpretation. In the final coded product dataset, plasmid prediction was positive for only 5/30 AMR-associated hits (16.7%), and no iMGE- or phage-associated ARG context was detected. This profile contrasts with the large survey by Tóth et al., where ARGs were detected in several commonly used probiotic species and, among ARG-positive samples, 66% were linked to plasmids or iMGEs, although no bacteriophage-linked ARGs were found [17]. It also appears less alarming than the earlier shotgun-based analysis of probiotic samples that detected more than 70 ARGs and numerous ARGs associated with plasmids, phages or iMGEs [16]. A similar AMR–resistance gene–mobile element perspective has recently been applied to *E. coli* from healthy farm animals, further supporting the relevance of mobilome-aware interpretation in veterinary AMR surveillance [32]. The limited proportion of predicted plasmid-associated ARGs in our farm-animal dataset supports a restrained conclusion: these products were not a prominent reservoir of high-priority mobile ARGs, but predicted plasmid contexts associated with tetracycline, aminoglycoside and macrolide/lincosamide determinants justify continued surveillance.

A further strength of the present work is that it avoids the common interpretive error of collapsing all AMR-associated signals into a single risk category. An intrinsic chromosomal determinant in a probiotic-associated *E. faecium* strain, a partial or nudged CARD hit on a short contig, and an acquired ARG predicted near a mobile element do not carry the same biological meaning. The present data therefore support a tiered framework: background or intrinsic determinants should be reported but not overinterpreted; acquired determinants without mobility evidence should trigger cautious interpretation; and acquired determinants with predicted plasmid, iMGE or phage context should be prioritized for confirmatory analysis. This framework is consistent with the broader horizontal gene transfer (HGT) literature, which emphasizes that mobility, ecological opportunity and recipient compatibility determine whether an ARG becomes a clinically relevant hazard [11,12,16,17].

The imperfect agreement between label information, viable recovery and sequencing results is also relevant. One labelled bacterial component was not recovered under the applied culture conditions, and one labelled *Pediococcus acidilactici* component was recorded as *Pediococcus pentosaceus*. These findings should be interpreted cautiously because culture conditions can bias recovery and because metagenomic detection may capture DNA from viable and non-viable cells. Nevertheless, they agree with previous reports that commercial probiotic products may require independent identity verification and strain-level confirmation in addition to ARG screening [18,26]. For feed-additive safety assessment, label concordance is not merely a quality-control issue; it determines which EFSA cut-offs, taxonomic assumptions and risk categories are appropriate.

This study has limitations that should be acknowledged transparently. First, the analysis relied on short-read sequencing and assembled contigs; therefore, predicted plasmid association or proximity to mobile elements should be interpreted as suggestive genomic context rather than experimental proof of transferability. Long-read sequencing, plasmid closure and conjugation experiments would be required to confirm whether specific ARGs are chromosomal, plasmid-borne or located within transferable composite elements. Thus, the present workflow should be viewed as a practical screening framework, with long-read sequencing reserved for confirmatory characterization of higher-priority or mobile-context-positive findings. Second, some CARD/RGI hits were nudged or based on partial coverage, and these should be treated as AMR-associated signals rather than definitive functional resistance determinants. Third, the MIC panel was broader than the EFSA core panel, meaning that some antimicrobial substances were useful for exploratory phenotypic profiling but not directly interpretable through probiotic-specific cut-offs. Finally, the dataset represents a targeted panel of selected commercially available farm-animal probiotic products rather than a statistically representative market-wide prevalence survey. Therefore, the findings should be interpreted as a focused safety assessment of relevant product types and as a model for integrated MIC–resistome–mobilome evaluation, not as an estimate of ARG prevalence across the entire farm-animal probiotic market. In addition, the pre-purchase storage and distribution history of commercial products could not be standardized, which may have influenced culture-based recovery of viable components. Therefore, culture-negative findings should not be extrapolated to all batches of the same product.

Despite these limitations, the study provides a practical and balanced model for veterinary probiotic safety assessment. The most defensible conclusion is that the investigated farm-animal probiotic products did not show evidence of high-priority mobile ARG carriage, while still containing AMR-associated determinants that require taxon-aware interpretation. This conclusion is more nuanced than both reassurance based solely on the absence of *vanA*/*vanB* or ESBL genes and alarm based solely on total ARG detection. For future product evaluation, we recommend an integrated workflow consisting of product metadata review, viability and identity confirmation, standardized MIC testing, WGS/metagenomic resistome analysis, explicit mobilome-context assessment and targeted long-read or transferability testing for higher-priority findings. Such a framework would be consistent with EFSA principles and with the emerging international literature on probiotic resistome surveillance.

## 4. Materials and Methods

### 4.1. Product Selection and Metadata Extraction

Commercially available probiotic products intended for farm-animal use were selected from the Hungarian/EU veterinary market based on declared use in poultry, swine, cattle or horses and the presence of at least one declared bacterial probiotic component. Products were obtained as commercially available preparations through regular veterinary/feed-additive distribution channels rather than through manufacturer-provided research batches. Label information was recorded directly from product packaging and, where available, accompanying product documentation. Products containing only fungal or yeast-based probiotic components were excluded from the final bacterial AMR/resistome analysis. Product metadata were extracted from labels and product documentation, including declared microbial species, strain designation, declared viable counts and target animal species. Products were pseudonymized as A–G throughout the manuscript to avoid commercial targeting while preserving scientific interpretability. The study was designed as a targeted exploratory safety assessment rather than as a market-wide prevalence survey. The final dataset was restricted to farm-animal products for which bacterial probiotic components could be evaluated by culture-based recovery and sequencing-based resistome analysis. The sample size therefore reflects the availability of eligible commercial farm-animal probiotic products within the screened Hungarian/EU market segment and the exclusion of products containing only fungal or yeast-based components. Consequently, the selected products should be interpreted as a focused panel of commercially relevant farm-animal probiotic preparations, not as a statistically representative sample of the entire probiotic feed-additive market.

The storage and distribution history before purchase was not experimentally controlled and may have differed among products. Therefore, culture-based recovery was interpreted as viability under the conditions encountered for the purchased product units and the applied laboratory recovery protocol, rather than as a definitive statement on viability across all batches or storage conditions. This limitation is particularly relevant for labelled components that were not recovered as viable cultures.

### 4.2. Culture-Based Recovery and Phenotypic MIC Testing

Where viable bacterial components were recovered, isolates were subjected to broth microdilution MIC profiling using two-fold antimicrobial dilutions in 96-well microtiter plates. Bacterial inocula were prepared from overnight cultures and added to antimicrobial-containing wells together with growth-positive and sterility-negative controls. Plates were incubated aerobically at 37 °C for 18–24 h. MIC values were read visually as the lowest antimicrobial concentration preventing visible growth. MIC interpretation was performed conservatively and in a taxon-aware manner. EFSA/FEEDAP microbiological cut-off concepts were considered where relevant for probiotic-associated taxa, whereas non-core or taxonomically non-informative agents were treated as exploratory phenotypic profiling markers rather than direct evidence of acquired resistance. Inequality MIC values were retained in the tables and approximated only for heatmap visualization.

Species-level identification of recovered isolates was confirmed by Matrix-Assisted Laser Desorption/Ionization Time-Of-Flight Mass Spectrometry (MALDI-TOF MS) according to the manufacturer’s (Bruker Daltonics, Bremen, Germany) instructions.

### 4.3. DNA Extraction and Sequencing

DNA was extracted from bacterial suspensions using the QIAamp DNA kit (Qiagen, Hilden, Germany) according to the manufacturer’s instructions. Paired-end short-read sequencing was performed on an Illumina NextSeq (Illumina, Inc., San Diego, CA, USA) platform. Short-read sequencing was selected because the primary objective was first-line resistome screening across multiple commercial product-derived samples rather than complete genome or plasmid closure. This approach provides sufficient resolution for assembled-contig-based ARG detection and comparative product-level screening, while recognizing that chromosomal versus plasmid localization and structural linkage of ARGs cannot be fully resolved without long-read sequencing. Long-read sequencing was therefore considered a confirmatory follow-up approach for higher-priority findings, especially ARGs predicted to be plasmid-associated or linked to mobile genetic elements.

### 4.4. Bioinformatic Processing and ARG Detection

Raw sequencing reads were quality checked using FastQC v0.11.9 [33] and filtered using Trim Galore v0.6.6 [34]. Filtered reads were assembled into contigs using MEGAHIT v1.2.9 [35]. Open reading frames were predicted with Prodigal v2.6.3 [36]. Predicted protein sequences were screened against the CARD using the RGI. Retained hits were evaluated according to CARD ARO assignment, percentage length of reference sequence, sequence identity, nudged/non-nudged status and taxonomic context. Hits classified as perfect or strict were prioritized for interpretation, whereas nudged or partial hits were treated as AMR-associated signals requiring cautious interpretation rather than definitive evidence of functional resistance.

RGI v6.0.5 was used with CARD version 4.0.1, downloaded on 29 May 2025. Only hits classified as perfect or strict, or nudged hits retained after manual review of percentage length of reference sequence, sequence identity and taxonomic context, were included in the final interpreted dataset.

### 4.5. Mobilome and Genomic-Context Analysis

Potential mobility of ARG-containing contigs was evaluated using MobileElementFinder v1.0.3 [14], PlasFlow v1.1 [37] and VirSorter v2.2.2 [38]. ARGs with predicted plasmid, iMGE or phage association were treated as higher-priority findings requiring cautious interpretation. Because short-read assemblies may not fully resolve plasmid or composite transposon structures, predicted mobile context was not considered proof of transferability.

### 4.6. Risk-Stratified Interpretation

ARG findings were classified using a conservative risk-stratified framework. Determinants repeatedly associated with the expected probiotic genus and lacking predicted mobile context were considered intrinsic/background or low-concern findings. Acquired determinants associated with clinically or veterinary-relevant antimicrobial classes were considered moderate-priority findings, particularly when plasmid-predicted. High-priority findings were predefined as mobile glycopeptide resistance genes, mobile colistin resistance genes, transferable oxazolidinone resistance genes, ESBL genes or carbapenemase genes.

### 4.7. Data Analysis and Visualization

Descriptive analyses were performed on product metadata, MIC values and ARG outputs. Gene frequencies, sample-level ARG counts, taxonomic assignment frequencies and predicted mobile-context counts were summarized. MIC values containing inequality symbols were retained in tables and approximated only for heatmap visualization.

## 5. Conclusions

The sampled commercial farm-animal probiotic products analyzed in this study did not contain detectable high-priority mobile ARGs such as *vanA*/*vanB*, *mcr*, *optrA*, *poxtA*, *cfr*, ESBL or carbapenemase genes. The resistome was dominated by recurrent taxon-associated background determinants, particularly in *Enterococcus*-containing products. However, the detection of acquired AMR-associated genes and occasional plasmid-predicted contexts demonstrates that probiotic feed additives should not be assessed by MIC data or genomic screening alone. A robust safety framework should integrate phenotypic MIC profiling, taxonomic confirmation, resistome analysis and mobilome-aware interpretation.

## Figures and Tables

**Table 1:** Commercial farm-animal probiotic products and labelled microbial components included in the study. CFU: colony forming unit.

Product	Labelled Microorganism (s)	Strain Designation(s)	Declared Concentration	Target Animal Species
A	*Enterococcus faecium*	NCIMB 10415 (C05808)	1 × 10^10^ CFU/g	poultry, swine, cattle
B	*Enterococcus faecium*	NCIMB10415 (4B1705)	1 × 10^8^ CFU/g	cattle
C	*Lactobacillus plantarum*	DSM12837	1 × 10^9^ CFU/g	horse
	*Pediococcus acidilactici*	DSM16243		
	*Enterococcus faecium*	DSM7134		
D	*Pediococcus acidilactici*	CNCM1-4622 (MA18/5M)	2.5 × 10^9^ CFU/g	poultry, swine
E	*Enterococcus faecium*	DSM7134	1 × 10^9^ CFU/g	swine
F	*Enterococcus faecium*	DSM7134	1 × 10^9^ CFU/g	swine
G	*Enterococcus faecium*	DSM7134 (401841)	1-10^11^ CFU/g	poultry, swine
	*Bacillus subtilis*	PB6 ATCC-PTA6737 (4B1823)	5 × 10^9^ CFU/kg	

**Table 2:** Minimum inhibitory concentration (MIC) values (µg/mL) for recovered bacterial isolates. The complete antimicrobial panel is visualized in Figure 1, and the full editable dataset is available from the corresponding author upon reasonable request. MIC values should be interpreted as taxon-aware safety-screening data. EFSA/FEEDAP microbiological cut-off values were considered where applicable, whereas Clinical and Laboratory Standards Institute (CLSI)/European Committee on Antimicrobial Susceptibility Testing (EUCAST) clinical breakpoints were not used as primary interpretive criteria for organism–drug combinations lacking validated probiotic- or species-specific clinical interpretation. 1. Penicillin; 2. Aminopenicillin; 3. Ceftriaxone; 4. Gentamicin; 5. Oxytetracycline; 6. Doxycycline; 7. Clindamycin; 8. Florfenicol; 9. Tilmicosin/tylosin; 10. Tiamulin; 11. Sulfonamide; 12. Trimethoprim; 13. Vancomycin.

Product	Labelled Organism	Recovered Organism	1	2	3	4	5	6	7	8	9	10	11	12	13
			µg/mL												
A	*Enterococcus faecium*	*Enterococcus faecium*	4	1	32	64	0.25	0.06	4	8	2	64	256	256	1
B	*Enterococcus faecium*	*Enterococcus faecium*	4	1	32	64	0.25	0.06	4	8	2	64	256	256	1
C	*Pediococcus acidilactici*	*Pediococcus pentosaceus*	1	16	32	32	32	16	0.03	8	4	128	256	256	64
	*Enterococcus faecium*	*Enterococcus faecium*	4	1	32	32	0.25	0.06	4	8	1	128	256	256	1
D	*Pediococcus acidilactici*	*Pediococcus acidilactici*	0.25	8	16	64	32	8	0.03	8	1	128	256	256	64
E	*Enterococcus faecium*	*Enterococcus faecium*	8	1	32	64	0.25	0.06	4	8	1	64	256	256	1
F	*Enterococcus faecium*	*Enterococcus faecium*	4	1	32	64	0.25	0.06	8	8	2	64	256	256	2
G	*Enterococcus faecium*	*Enterococcus faecium*	0.5	0.125	1	0.5	0.25	0.06	0.5	2	0.5	128	256	256	1
	*Enterococcus faecium*	*Enterococcus faecium*	2	1	1	1	0.125	0.06	0.5	2	0.25	128	64	0.5	0.25
	*Bacillus subtilis*	*Bacillus subtilis*	0.03	1	16	2	16	0.25	0.25	2	0.25	128	16	0.125	64

**Table 3:** Antimicrobial resistance (AMR)-associated CARD antibiotic-resistant organism (ARO) hits detected in the final coded product dataset and their working risk interpretation. Predicted plasmid context was treated as a risk modifier and not as experimental evidence of transferability. ARG: antimicrobial resistance gene.

ARG/ARO Hit	Hits	Dominant Taxon	Predicted Context	Interpretation
*aac* *(6′)-Ii*	6	*Enterococcus*	no predicted plasmid/iMGE/phage context	background/intrinsic or low concern unless mobile context is confirmed
*eatAv*	6	*Enterococcus*	no predicted plasmid/iMGE/phage context	background/intrinsic or low concern unless mobile context is confirmed
*msrC*	6	*Enterococcus*	no predicted plasmid/iMGE/phage context	background/intrinsic or low concern unless mobile context is confirmed
*tetM*	2	*Staphylococcus*	plasmid-predicted: 1	acquired/resistance-associated; interpret with identity, coverage and genomic context
*sat-4*	2	*Staphylococcus*/*Streptococcus*	no predicted plasmid/iMGE/phage context	acquired/resistance-associated; interpret with identity, coverage and genomic context
*aadA27*	1	*Acinetobacter*	no predicted plasmid/iMGE/phage context	acquired/resistance-associated; interpret cautiously because it was a single low-frequency hit
*tetS*	1	Bacilli	plasmid-predicted: 1	acquired/resistance-associated; interpret with identity, coverage and genomic context
*ermB*	1	Bacteria	plasmid-predicted: 1	acquired/resistance-associated; interpret with identity, coverage and genomic context
*aad(6)*	1	*Staphylococcus*	no predicted plasmid/iMGE/phage context	acquired/resistance-associated; interpret with identity, coverage and genomic context
*aph* *(3′)-IIIa*	1	*Streptococcus*	no predicted plasmid/iMGE/phage context	acquired/resistance-associated; interpret with identity, coverage and genomic context
*aph* *(3′)-Ia*	1	Bacteria	plasmid-predicted: 1	acquired/resistance-associated; interpret with identity, coverage and genomic context
*tet(W/N/W)*	1	*Streptococcus*	plasmid-predicted: 1	acquired/resistance-associated; interpret cautiously because of single-hit detection
*efmA*	1	*Enterococcus*	no predicted plasmid/iMGE/phage context	background/intrinsic or low concern unless mobile context is confirmed

## Data Availability

The sequencing data generated in this study have been deposited in the NCBI BioProject repository under accession PRJNA1461228 (BioSample submission: SUB16162299). The complete CARD/RGI output is provided as Supplementary Table S1. The remaining processed data are available from the corresponding author upon reasonable request.

## Supplementary Materials

The following supporting information can be downloaded at: https://www.mdpi.com/article/10.3390/antibiotics15060544/s1, Table S1: Complete CARD/RGI antimicrobial resistance gene (ARG) output with taxonomic assignment and predicted genomic context.

## Abbreviations

AMR	Antimicrobial resistance
ARG	Antimicrobial resistance gene
ARO	Antibiotic Resistance Ontology
CARD	Comprehensive Antibiotic Resistance Database
EFSA	European Food Safety Authority
FEEDAP	Feed Additives and Products or Substances
iMGE	Insertion sequence/mobile genetic element
MIC	Minimum inhibitory concentration
NGS	Next-generation sequencing
RGI	Resistance Gene Identifier
WGS	Whole-genome sequencing
